# Therapeutic Benefits of Nano-Echinacea Extract on Reproductive Injury Induced by Polystyrene Plastic Materials in Rat Model via Regulating Gut–Brain Axis

**DOI:** 10.3390/ijms26136097

**Published:** 2025-06-25

**Authors:** Yi-Yuh Hwang, Sabri Sudirman, Pei-Xuan Tsai, Chine-Feng Mao, Athira Johnson, Tai-Yuan Chen, Deng-Fwu Hwang, Zwe-Ling Kong

**Affiliations:** 1 Department of Food Science, National Taiwan Ocean University, Keelung 20224, Taiwan; 2graceyy@gmail.com (Y.-Y.H.); 11032040@email.ntou.edu.tw (P.-X.T.); pioneer@directintaiwan.com.tw (C.-F.M.); tychen@mail.ntou.edu.tw (T.-Y.C.); 2 Department of Fisheries Product Technology, Faculty of Agriculture, Universitas Sriwijaya, Indralaya 30662, Indonesia; sabrisudirman@unsri.ac.id; 3 Department of Polymer Science and Rubber Technology, Cochin University of Science and Technology, South Kalamassery 682002, India; athirajohnson07@gmail.com

**Keywords:** chitosan-silica nanoparticles, *Echinacea purpurea* extract, gut–brain axis, hypothalamus–pituitary–gonadal axis, male fertility, polystyrene nanoplastics

## Abstract

Plastics pollution is a critical global environmental issue, with growing concern over the increasing presence of nanoplastic particles. Plastics are major environmental pollutants that adversely affect human health, particularly when plastics from food sources enter the body and pose potential risks to reproductive health. *Echinacea purpurea* is an immunologically active medicinal plant containing phenolic acids and alkylamides. Nanoparticles present a promising approach to enhance the effectiveness, stability, and bioavailability of *Echinacea purpurea* ethanol extract (EE) active components. This study aimed to determine the protective effects of chitosan-silica-*Echinacea purpurea* nanoparticles (CSE) against reproductive injury induced by polystyrene nanoplastics (PS-NPs) in male rats. The results showed that CSE dose-dependently reduced oxidative damage and protected intestinal and reproductive health. Furthermore, CSE improved gut microbiota dysbiosis, preserved barrier integrity, and attenuated PS-NPs-induced inflammation in the colon, brain, and gonads. Inflammatory factors released from the gut can enter the bloodstream, cross the blood–brain barrier, and potentially modulate the hypothalamic–pituitary–gonadal (HPG) axis. CSE has also been shown to elevate neurotransmitter levels in the colon and brain, thereby repairing HPG axis dysregulation caused by PS-NPs through gut–brain communication and improving reproductive dysfunction. This study enhances our understanding of CSE in modulating the gut–brain and HPG axes under PS-NPs-induced damage. CSE demonstrates the capacity to provide protection and facilitate recovery by mitigating oxidative stress and inflammation, restoring gut microbiota balance, and preserving hormone levels in the context of PS-NPs-induced injury.

## 1. Introduction

The pervasiveness of plastic pollution extends beyond its environmental impact and has infiltrated our primary sources of sustenance. A growing concern is the reproductive health risks associated with nanoplastics (NPs) derived from food sources, which have emerged as a significant challenge. Understanding and minimizing the reproductive injury caused by NPs is crucial as long as the consumption of plastic-contaminated food continues [1]. Therefore, the primary objective of this work is to investigate the protective effects of nano *Echinacea* ethanol extract on reproductive injury induced by polystyrene nanoplastics (PS-NPs) in male rats. This study provides vital information about ways to guard against the harmful effects of plastic pollution linked to food.

Male infertility is a serious reproductive health problem, and studies revealed that male reproductive issues account for approximately 50% of all occurrences of childlessness, affecting 8 to 12% of couples of childbearing age worldwide [2,3]. Furthermore, concerning research indicates a decline in typical sperm concentration and count among young Chinese men in recent years [4]. One of the main reasons behind this is the consumption of food containing microplastics. Animals unintentionally consume NPs, particularly when they ingest food with poor nutritional content, leading to the transfer of these particles into the food chain [5,6]. Upon ingestion, NPs enter the animal’s body, causing damage to the intestines and accumulating in various tissues and organs. Notably, smaller NPs can bypass barrier systems and accumulate in delicate organs such as the brain and gonads [7,8].

The reproductive systems of aquatic organisms are adversely affected by exposure to NPs [1]. For example, disruptions to the hypothalamic–pituitary–testicular axis in medaka (*Oryzias latipes*) can lead to abnormal blood levels of sex hormones and reproductive endocrine disorders, resulting in abnormal germ cell proliferation [9,10]. Studies have also demonstrated that the consumption of NPs by marine species causes not only physical and mechanical harm but also inhibits the digestive tract, slows growth, and prevents enzyme production [11,12]. In mammals, exposure to NPs has been closely linked to damage to sperm and testis in mice [13]. Sperm quality is a critical determinant of male fertility and is characterized by factors such as sperm quantity and deformity index [14]. NPs exposure significantly reduced spermatogenic cell count, disrupted spermatogenic cell organization, increased abnormality rates, and lowered the activity of sperm metabolism-related enzymes, such as succinate dehydrogenase and lactate dehydrogenase [13]. NPs not only cause risks to reproductive health but also cause detrimental effects on intestinal health. Studies have demonstrated that NPs disturb the intestinal microbiota, reduce the probiotic/pathogenic bacteria ratio, impair intestinal mucus secretion, harm the mucosal epithelium, and compromise the integrity of the intestinal barrier [15,16,17]. Additionally, commercial fish and deep-sea fish have been found to contain NPs in their stomachs and intestines, indicating that these particles are ingested through the food chain [18,19]. While limited data exist for mammals, studies have shown that orally consumed NPs can be found in the gastrointestinal tract of mice, leading to inflammation and metabolic disorders [20,21]. It is generally recognized that smaller-sized plastics pose greater hazards to organisms due to their higher bioavailability and longer residence time in the body [22,23].

*Echinacea purpurea* (Asteraceae) is a medicinal herb renowned for its immunostimulatory effects and is widely used in various forms, such as tinctures, capsules, and infusions, to boost the immune system [24]. Extensive research has identified phenolic acids and isobutylamides as the bioactive components present in *Echinacea purpurea* ethanol extract (EE) [25]. Among these bioactive compounds, cichoric acid, a caffeic acid derivative with adjacent hydroxyl groups on its phenolic ring, is responsible for the radical scavenging capacity of EE [26]. Furthermore, EE exhibits a broad range of pharmacological activities, including anti-inflammatory, antimicrobial, antiviral, and antioxidant properties, making it valuable for treating wounds, the common cold, coughs, bronchitis, and upper respiratory infections [27,28,29]. Despite its diverse therapeutic potential, the biomedical application of EE is limited by its astringent and bitter taste, as well as its low aqueous solubility and oral bioavailability [30]. Additionally, the direct utilization of phenolic compounds is challenged by their rapid release, susceptibility to environmental stress, and poor permeation [31]. To overcome these limitations, the use of nanocarriers for nanoencapsulation has emerged as a promising solution. Notably, studies have successfully encapsulated EE within chitosan-silica nanoparticles (CS), which exhibit antioxidant and anti-inflammatory properties in vitro [25]. These nanocarriers act as regulated drug delivery systems, enhancing the oral bioavailability of lipophilic and hydrophobic drugs, including the bioactive constituents of EE.

Chitosan is a nontoxic, biodegradable and biocompatible polysaccharide having a cationic charge on the surface. Chitosan is obtained by partial or complete deacetylation of chitin and contains a hydrophobic backbone and amino groups at the C2 position. It acts as a biocatalyst during the silicification process. Mesoporous silica nanoparticles are well known for their controlled porous structure, high surface area, controlled drug delivery, high surface area and nontoxicity. They are also suitable for conjugation due to the presence of a silanol group on the surface [32,33].

The cumulative evidence highlighting the reproductive and health risks associated with PS-NPs derived from food sources necessitates a comprehensive understanding of their impact and the development of effective preventive measures. Therefore, this work aimed to explore the protective effects of chitosan-silica-*Echinacea purpurea* extract nanoparticles (CSE) on reproductive injury induced by PS-NPs in male rats. Also, histopathological studies of the colon have been conducted to evaluate the anti-inflammatory activities of CSE. By investigating the potential benefits of this extract, we can gain critical insights into mitigating the adverse consequences of food-related plastic pollution and contribute to safeguarding reproductive health.

## 2. Results

### 2.1. FTIR Analysis

**Figure 1:**
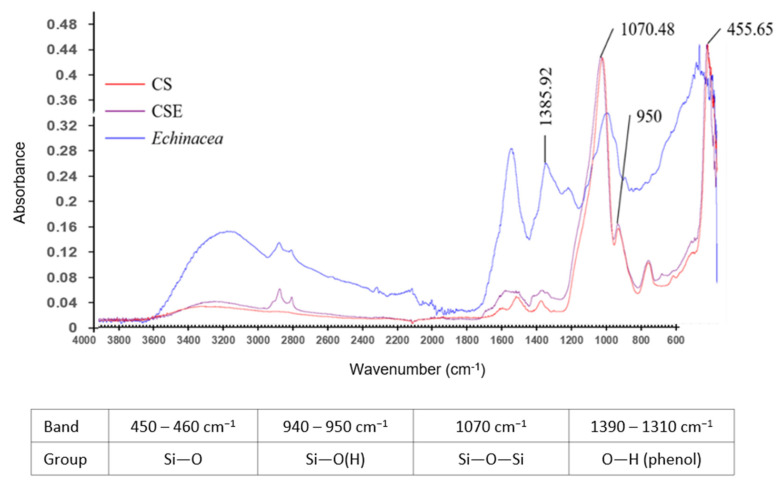
Fourier-transform infrared spectra (FTIR) of CS, CSE, and *Echinacea purpurea* ethanol extract (EE). CS, chitosan-silica nanoparticles; CSE, chitosan-silica-*Echinacea purpurea* extracts composite nanoparticles.

Fourier Transform Infrared Spectroscopy (FTIR) analysis of the chitosan-silica nanoparticles (CS), *Echinacea purpurea* ethanol extract (EE), and chitosan-silica-*Echinacea purpurea* nanoparticles (CSE) is shown in Figure 1. The results showed that the EE had a bending vibration of the OH bond in the polyphenolic group at 1385.92 cm^−1^ [34]. The CS-EE conjugate exhibited absorption bands of the stretching vibrations of the siloxane (Si-O-Si) group at 1070 cm^−1^, the Si-O(H) group at 950 cm^−1^, and the Si-O group at 455 cm^−1^ [35,36]. The FTIR spectra indicated that CS had absorption bands of both the amino group of chitosan and the siloxane and Si-O(H) groups, suggesting the formation of CS nanoparticles by the co-polymerization of chitosan and silica dioxide. The absorption peaks of CSE and CS were almost identical, with no obvious signal of EE, indicating that CS could effectively encapsulate the sample as a good nanocarrier for drug delivery.

### 2.2. DLS Analysis of Nanoparticles

The average particle size and interface potential of CS and CSE were measured using a Dynamic Light Scattering (DLS) instrument. The average particle size of CS and CSE was 101.1 ± 18.3 nm and 163.8 ± 30.5 nm, respectively, indicating an increase in particle size after loading EE into CS nanoparticles. Particles with a diameter of less than 500 nm can be engulfed by cells, facilitating intracellular medication delivery [37]. The interface potential, which represents the electrical charge surrounding colloidal particles, is commonly assessed using a threshold of +30 or −30 mV to determine the stability of colloidal suspensions. The interface potential values for CS and CSE were determined to be −29.0 ± 0.34 mV and −25.5 ± 1.39 mV, respectively. The biosilicification method enables the co-aggregation of positively charged chitosan and negatively charged silica nanoparticles via electrostatic forces, resulting in the formation of CS nanoparticles, which can simultaneously enhance bioavailability and reduce cytotoxicity [38].

### 2.3. Level of Biomarkers for Liver Damage

**Figure 2:**
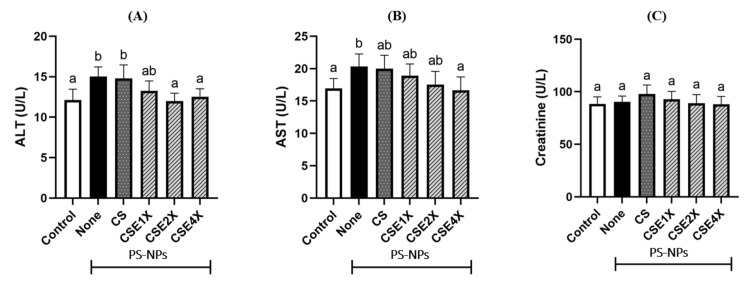
Effects of CS and CSE on the (**A**) alanine aminotransferase (ALT), (**B**) aspartate aminotransferase (AST), and (**C**) creatinine levels in plasma of rats after 8 weeks of treatments. The data are expressed as mean ± SD (n = 7). The values with different letters (a and b) represent significantly different (*p* < 0.05) as analyzed by the Tukey test. CS, chitosan-silica nanoparticles; CSE, chitosan-silica-*Echinacea purpurea* extracts composite nanoparticles.

Alanine aminotransferase (ALT) and aspartate aminotransferase (AST) are commonly used biomarkers for liver damage. Elevated ALT and AST levels were observed in the None group compared to the Control group (Figure 2). However, the levels of ALT and AST reduced significantly after CSE treatment, especially at high doses of CSE (CSE4X). Creatinine level was higher in the CS group followed by CSE1X. But there is no significant difference between all groups. Creatinine level was higher in the CS group followed by CSE1X. However, there is no significant difference between all groups.

### 2.4. Antioxidant Properties of CSE

**Figure 3:**
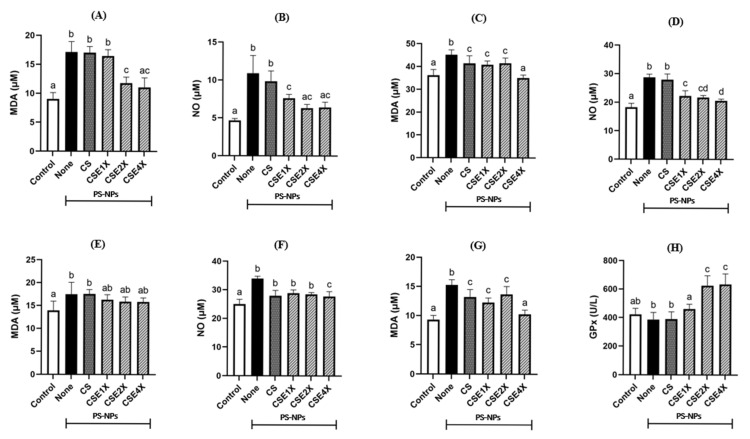
Effects of CSE with different dosages on the malondialdehyde (MDA) and nitric oxide (NO) levels in colon (**A**,**B**), brain (**C**,**D**), testis (**E**,**F**), MDA level and GPx activity (**G**,**H**) in plasma of rats after 8 weeks of treatments. The data are expressed as mean ± SD (n = 7). The values with different letters (a–d) represent significantly different (*p* < 0.05) as analyzed by the Tukey test.

To evaluate the changes in oxidative stress, MDA and GPx (Figure 3) levels in the body of rats were analyzed. The findings demonstrated that exposure to PS-NPs resulted in a substantial increase in oxidative stress, as indicated by elevated MDA levels, signifying heightened lipid peroxidation. As shown in Figure 3, the MDA level was lower in CSE4X, and it has no significant difference from the Control group. In the colon also, the lowest MDA level was observed in the CSE4X group followed by the CSE2X group, which was slightly higher than the Control group. A decrease in MDA level was observed in both brain and testis. However, in the brain, all of the CSE group exhibited the same level of MDA, which was slightly higher than the Control. Additionally, there was a significant increase in GPx activity compared to the Control group, suggesting a compromised antioxidant defense system. Both CSE2X and CSE4X showed high GPx levels compared to the CSE1X group. These results collectively point to PS-NPs-induced oxidative stress in rats.

The elevated NO concentration in colon, brain, and testes when expose by PS-NPs (None group). In colon and brain, treatment with various doses of CSE significantly reduced the NO compared to the control group. In testes, there is no significant difference in NO level between CS, CSE1X, and CSE2X groups. However, high-dose of CSE (CSE4X) significantly decreased the NO level.

### 2.5. Effect of CSE on Hypothalamus–Pituitary–Gonadal (HPG) Axis and Sperm Metabolism

**Figure 4:**
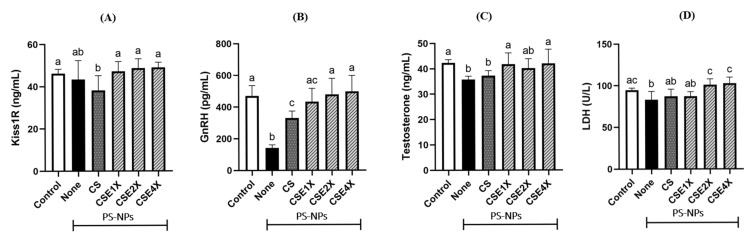
Effects of CSE with different dosages on the (**A**) Kiss1 receptor (Kiss1R), (**B**) gonadotropin-releasing hormone (GnRH), (**C**) testosterone levels, and (**D**) lactate dehydrogenase (LDH) activity in plasma of rats after 8 weeks of treatments. Data are expressed as mean ± SD (n = 7). The values with different letters (a–c) represent significantly different (*p* < 0.05) as analyzed by the Tukey test.

The present research sought to elucidate the underlying mechanisms within the HPG axis that contribute to the reproductive damage caused by PS-NPs (Figure 4A–C). PS-NPs exposure resulted in reduced Kiss1R, GnRH, and testosterone levels. In Figure 4, the Kiss1R level was lower in the None and CS groups compared to others. The GnRH level was higher in the CSE4X group, followed by the CSE2X and Control groups. There is no significant difference between the LH levels of the CSE2X and CSE4X groups. The testosterone level was higher in the CSE4X, CSE1X, and Control groups when compared to others.

To further investigate the spermatozoa of male rats, the enzyme lactate dehydrogenase (LDH) involved in sperm metabolism has been studied. The results revealed a substantial reduction in LDH levels among the PS-NPs groups compared to the Control group (Figure 4D), indicating inadequate energy supply to sperm, impeding energy metabolism, and resulting in aberrant sperm production [13]. However, the administration of CSE exhibited a remarkable dose-dependent restoration of LDH expression, reaching levels comparable to those of the Control group.

### 2.6. Histopathological Changes in Seminiferous Tubule (ST)

**Figure 5:**
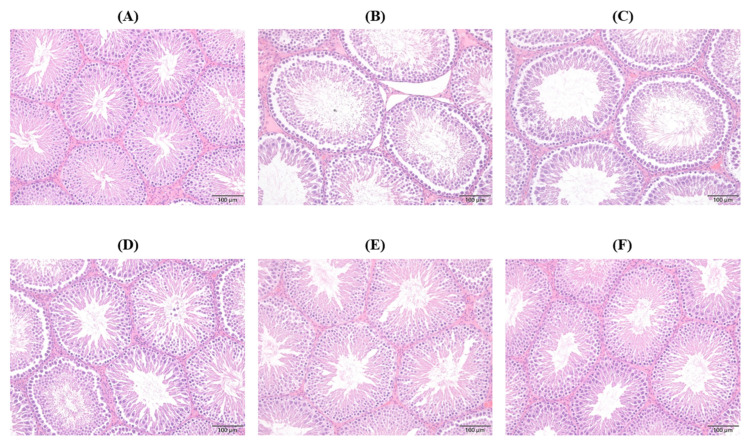
Effects of CSE with different dosages on seminiferous tubules (ST) of testis in rats after 8 weeks of treatment: (**A**) Control group, (**B**) PS-NPs group: 5 mg/kg PS-NPs, (**C**) CS group: 5 mg/kg PS-NPs + 372 mg/kg CS, (**D**) CSE1X group: 5 mg/kg PS-NPs + 93 mg/kg CSE, (**E**) CSE2X group: 5 mg/kg PS-NPs + 186 mg/kg CSE, and (**F**) CSE4X group: 5 mg/kg PS-NPs + 372 mg/kg CSE. Representative images of hematoxylin and eosin (H&E) sections in the testis of each group.

The testicular morphology of the Control group showed normal testicular features, characterized by intact seminiferous tubule (ST) structure and Leydig cells (Figure 5). All spermatogenic cells, including the spermatogonia, primary spermatocytes, and early spermatids, were intact and mature sperms filled the lumen, with a normal density of interstitial connective tissue (Figure 5A). On the other hand, PS-NPs cause severe edema around the ST, loss of the epithelium, thinning of the tubule wall, increased luminal area, and severe vacuolization inside the ST. There was an obvious decrease in the number of all spermatogenic cells, with abnormally large and deeply stained spermatogonia (Figure 5B). The pathological changes in the CS group were similar to those in the PS-NPs group (Figure 5C). After treatment with CSE, the structure of the seminiferous tubules improved in a dose-dependent manner, with reduced irregularity of the epithelium, normal arrangement of reproductive cells, and intact interstitial connective tissue (Figure 5D–F). Some vacuolization, loss of spermatocytes, and some damage in the epithelium surrounding the ST were still observed in the testis of CSE1X and CSE2X-treated rats (Figure 5D,E). However, almost normal ST structures with intact spermatogenic cells and no edema were observed in the testis of the CSE4X group (Figure 5F).

### 2.7. Microbial Diversity in the Gut

**Figure 6:**
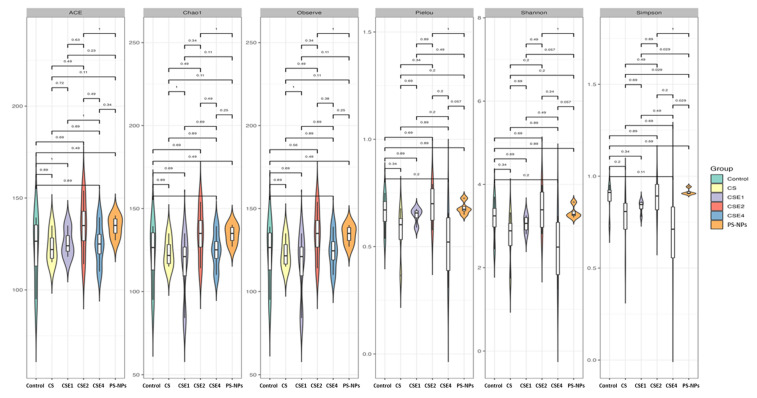
Effect of PS-NPs, CS, and CSE treatments on the microbial abundance in rat fecal microbiota. Alpha diversity, measured by Abundance-based Coverage Estimator (ACE), Chao1 estimator, observed species richness, Pielou’s evenness, Shannon index and Simpson index are plotted. Metagenomic analysis was performed on four biological samples randomly selected from each group. Treatment groups: Control (without any treatment), PS-NPs (5 mg/kg PS-NPs), CS (5 mg/kg PS-NPs + 372 mg/kg CS), CSE1X (5 mg/kg PS-NPs + 93 mg/kg CSE), CSE2X (5 mg/kg PS-NPs + 186 mg/kg CSE), and CSE4X (5 mg/kg PS-NPs + 372 mg/kg CSE).

To investigate the variations in structural diversity of gut microbiota between the PS-NPs group and CSE groups, we evaluated microbial alpha diversity using the Chao1, Simpson, and Shannon indexes. These indices provide estimates of richness (the number of different species), evenness (species distribution), and overall diversity, respectively [39]. Notably, there were no significant differences observed in alpha diversity, suggesting that PS-NPs did not have a noticeable impact on the microbial diversity within individuals (Figure 6).

**Figure 7:**
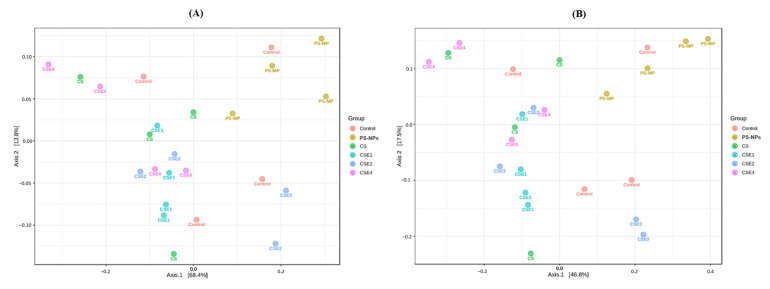
Effect of PS-NPs, CS, and CSE treatments of the principal coordinate analysis (PCoA) in rat. Beta diversity based on (**A**) Weighted UniFrac distances (PERMANOVA R = 0.4359, *p* = 0.001) and (**B**) Bray–Curtis’s dissimilarity (PERMANOVA R = 0.4831, *p* = 0.002). Metagenomic analysis was performed on four biological samples randomly selected from each group. Treatment groups: Control (without any treatment), PS-NPs (5 mg/kg PS-NPs), CS (5 mg/kg PS-NPs + 372 mg/kg CS), CSE1X (5 mg/kg PS-NPs + 93 mg/kg CSE), CSE2X (5 mg/kg PS-NPs + 186 mg/kg CSE), and CSE4X (5 mg/kg PS-NPs + 372 mg/kg CSE).

In terms of beta diversity, which characterizes dissimilarities between species communities, we employed principal coordinate analysis (PCoA) based on weighted UniFrac distances and Bray–Curtis’s dissimilarity in each group (Figure 7). These measures enabled the assessment of species composition similarities across samples. Interestingly, the gut microbiome composition profiles of the PS-NPs group exhibited substantial dissimilarities when compared to those of the CSE-treated groups, as evidenced by both the weighted UniFrac distances (Figure 7A) and Bray–Curtis’s dissimilarity (Figure 7B).

Exposure to PS-NPs did not affect microbial species richness (α diversity). However, there were significant differences in microbial structure (β diversity) between the groups, suggesting that the doses used in this study did not substantially alter the overall abundance of microbial species in the individuals, in agreement with previous observations [40].

### 2.8. Microbial Composition in Gut

**Figure 8:**
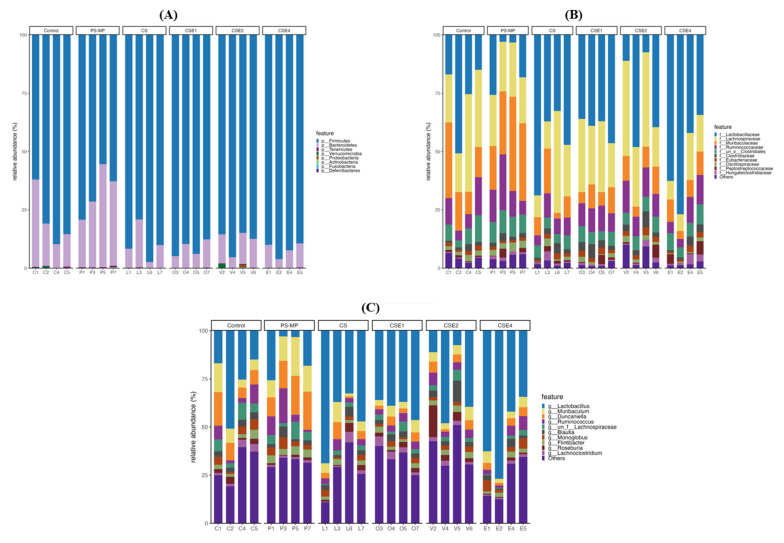
Relative gut microbiota abundance at the (**A**) phylum, (**B**) family, and (**C**) genus levels. The histogram of species distribution at the phylum, family or genus levels in each group revealed by 16S rRNA sequencing (different colors represent different bacteria at each level). Metagenomic analysis was performed on four biological samples randomly selected from each group. Treatment groups: Control (without any treatment), PS-NPs (5 mg/kg PS-NPs), CS (5 mg/kg PS-NPs + 372 mg/kg CS), CSE1X (5 mg/kg PS-NPs + 93 mg/kg CSE), CSE2X (5 mg/kg PS-NPs + 186 mg/kg CSE), and CSE4X (5 mg/kg PS-NPs + 372 mg/kg CSE).

To investigate the impact of PS-NPs on the gut–brain axis, we analyzed the changes in gut microbiota composition. The gut is one of the most crucial tissues that is directly influenced by NPs. The development of the gut immune system relies on gut microbiota, and the balance of gut microbiota can modulate inflammatory responses [41]. The results illustrate the species annotation analysis at the phylum, class, order, family, genus, and species levels using 16S rRNA sequencing. The figures display only the top ten abundant bacterial species, while the remaining species are grouped as “Others.” *Firmicutes* and *Bacteroidetes* are the two predominant phyla in the gut microbiota of rats, and their relative abundances were compared among different groups (Figure 8A). The PS-NPs group exhibited a decrease in *Firmicutes* and an increase in *Bacteroidetes* compared to the Control group. Conversely, the CSE groups showed similar gut microbiota composition to the Control group.

Analyzing the family level composition, the PS-NPs group exhibited a significant decrease in *Lactobacillaceae* compared to the Control group. In contrast, high-dose CSE treatment resulted in an increased proportion of *Lactobacillaceae* and a decrease in the abundance of inflammation-associated *Lachnospiraceae* (Figure 8B). These findings suggest that CSE has the potential to improve the gut microbiota profile by promoting the growth of beneficial *Lactobacillaceae* and reducing the presence of detrimental *Lachnospiraceae*. *Lachnospiraceae* has been implicated in various health conditions, including metabolic syndrome, obesity, diabetes, liver diseases, IBD, depressive syndromes, and multiple sclerosis syndrome [42].

At the genus level, the gut microbiota composition changed after PS-NPs exposure (Figure 8C). The PS-NPs group exhibited a significant decrease in *Lactobacillus* compared to the Control group. Conversely, the CSE-treated group, particularly the CSE4X group, showed a significant increase in *Lactobacillus,* which is renowned for its anti-inflammatory properties, characterized by the suppression of proinflammatory cytokines, and improved integrity of the intestinal epithelial barrier [43].

### 2.9. Effect of CSE on Inflammatory Cytokines

**Figure 9:**
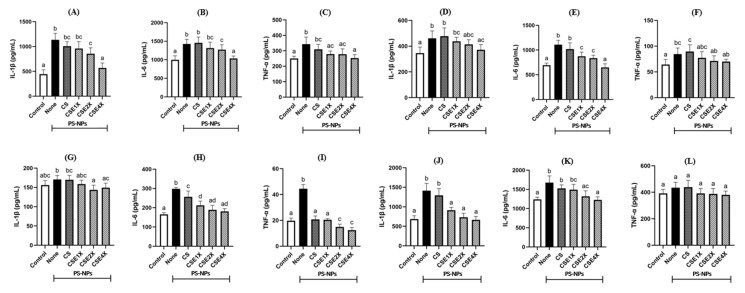
Effects of CSE with different dosages on the interleukin-1β (IL-1β), IL-6, and tumor necrosis factor-α (TNF-α) levels in testis (**A**–**C**), colon (**D**–**F**), plasma (**G**–**I**), and brain (**J**–**L**) of rats after 8 weeks of treatments. The data are expressed as mean ± SD (n = 7). The values with different letters (a–d) represent significantly different (*p* < 0.05) as analyzed by the Tukey test.

The findings demonstrated a significant reduction in the elevated levels of inflammatory cytokines, including interleukin (IL)-1β, IL-6, and tumor necrosis factor-α (TNF-α), induced by PS-NPs in the testes upon treatment with CSE (Figure 9A–C). The IL-1β, IL-6, and TNF-α were higher in the PS-NPs group when compared to other groups. The level of IL-1β, IL-6, and TNF-α groups were highly reduced in both Control and CSE4X groups. The mitigating effect of CSE on IL-1β in the testicular tissue was found to be dose-dependent. Similar alterations in the inflammatory response were observed in the colon (Figure 9D–F), plasma (Figure 9G–I), and brain (Figure 9J–L) when compared to the testes. In colon, IL-1β, IL-6, and TNF-α were significantly higher in None and CS groups and highly reduced in the CSE4X group. In plasma also, IL-1β, IL-6, and TNF-α were lower in CSE-treated groups. It is noteworthy that the levels of TNF-α in the brain remained unaffected, while substantial variations were observed for IL-1β and IL-6. In contrast, they are significantly reduced when treated using CSE2X and CSE4X.

### 2.10. Histopathological Changes in Colon

**Figure 10:**
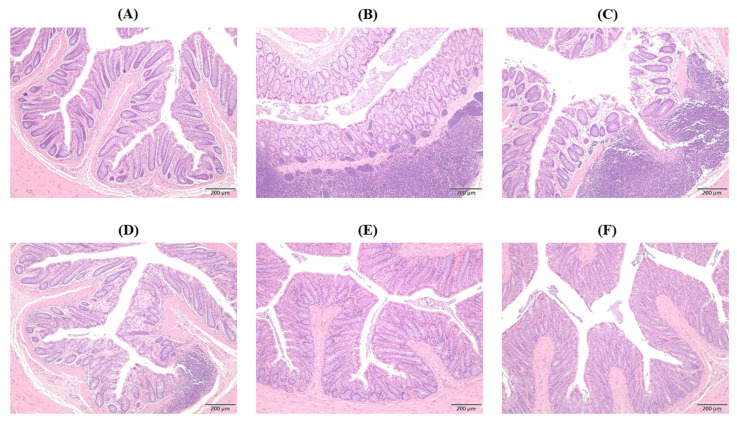
Effects of CSE with different dosages on colon in rats after 8 weeks of treatment: (**A**) Control group, (**B**) PS-NPs group: 5 mg/kg PS-NPs, (**C**) CS group: 5 mg/kg PS-NPs + 372 mg/kg CS, (**D**) CSE1X group: 5 mg/kg PS-NPs + 93 mg/kg CSE, (**E**) CSE2X group: 5 mg/kg PS-NPs + 186 mg/kg CSE, and (**F**) CSE4X group: 5 mg/kg PS-NPs + 372 mg/kg CSE. Representative images of hematoxylin and eosin (H&E) sections in the testis of each group.

Through histological analysis, hematoxylin and eosin (H&E)-stained sections demonstrate notable alterations in colon tissue caused by different treatments, indicating the anti-inflammatory effectiveness of CSE (Figure 10). Compared to the Control group (Figure 10A), specimens from the PS-NPs group (Figure 10B) showed substantial impairment of the regular structure of the colon, widespread ulceration, and inflammation influencing all layers of the gut wall, and it was almost similar to the CS group (Figure 10C). However, the neutrophilic inflammatory infiltrate is moderately reduced in CSE-treated colonic specimens, which show a remarkable restorative effect (Figure 10D–F).

### 2.11. Effect of CSE on Neurotransmitter Level

**Figure 11:**
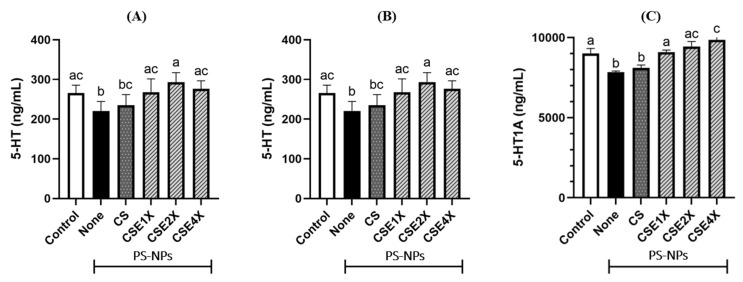
Effects of CS and CSE on the (**A**) 5-hydroxytryptamine (5-HT) level in colon, (**B**) 5-HT level in brain, and (**C**) 5-hydroxytryptamine receptor 1A (5-HT1A) level in brain of rats after 8 weeks of treatments. The data are expressed as mean ± SD (n = 7). The values with different letters (a–c) represent significantly different (*p* < 0.05) as analyzed by the Tukey test.

The present experiment examined the changes in 5-hydroxytryptamine (5-HT) levels in the colon (Figure 11A) and brain (Figure 11B), as well as alterations in the 5-hydroxytryptamine receptor 1A (5-HT1A receptor) to investigate the potential role of CSE treatment in attenuating PS-NPs-induced damage via the gut–brain axis (Figure 11C). The findings of the study demonstrated a significant elevation in the levels of the neurotransmitter 5-HT in both the colon and brain following treatment with CSE, effectively restoring them to normal levels comparable to the Control group. The colon exhibited more pronounced changes in 5-HT levels compared to the brain. Moreover, the expression of the 5-HT1A receptor in the brain was significantly attenuated due to the administration of PS-NPs, whereas CSE administration exhibited a notable improvement in this regard. The 5-HT1A receptor level was higher in the CSE4X group, followed by Control and CSE2X groups.

## 3. Discussion

Polystyrene nanoplastics (PS-NPs) can cause impaired liver function, characterized by significant increases in serum ALT and AST levels. A previous study reported that AST is also present in cardiac and skeletal muscle and also in erythrocytes, making ALT the most specific marker for liver damage [44]. Elevated serum ALT levels indicate a high specificity and a reasonable sensitivity for liver injury and are associated with an increased risk of liver-specific mortality as well as non-hepatic diseases such as type-2 diabetes mellitus, metabolic syndrome, cardiovascular diseases, and malignancies [45]. The levels of ALT and AST reduced significantly after CSE treatment, indicating that the sample dosages utilized in this study were within the rat’s tolerable range and had a liver-protective effect. The results also established the in vivo non-toxicity of CS nanoparticle carriers [38,46]. However, there was little potential for improvement in the observed damage due to the absence of loaded EE samples.

The results provide compelling evidence for the efficacy of the CSE sample as an antioxidant agent. The ability of CSE to decrease oxidative damage in a dose-dependent manner confirms its antioxidant effectiveness. By effectively reducing oxidative stress, the CSE sample may contribute to the preservation and maintenance of intestinal and gonadal health. Based on previous research, *Echinacea purpurea* extract has been found to contain abundant caffeic acid and its derivatives, as well as cichoric acid, which exhibits excellent antioxidant activity and serves as an effective free radical scavenger [47].

Damage to the testicular interstitial tissue by PS-NPs interferes with the LH-stimulated synthesis of testosterone by Leydig cells, resulting in decreased testosterone levels. This reduction in testosterone triggers corresponding signals to the hypothalamus, leading to increased LH release from the pituitary gland [48]. This cyclic process disrupts the balance of the HPG axis and subsequently affects the normal production of testosterone. Reduced testosterone level resulting from NPs exposure is regarded as a main contributor to decreased sperm quantity and epithelium height, connected to anti-androgenic properties of NPs [49,50]. Previous studies have shown the variations in the levels of reproductive endocrine hormones, including Kiss1R, GnRH, LH, and testosterone, within the HPG axis, in rat models [48,51]. The hormone decline in the HPG axis is also likely caused by PS-NPs-induced oxidative stress and inflammation. The higher levels of Kiss1R, GnRH, and testosterone in the CSE groups suggest a protective effect against PS-NPs-induced hormone disruptions. Antioxidative and anti-inflammatory properties of CSE mitigate oxidative stress and inflammation, respectively. Furthermore, CSE modulates the HPG axis, facilitating hormone release and restoring normal hormone levels for improved reproductive health. Existing literature supports the protective effects of CSE on hormone regulation and reproductive function [52]. CSE also has a protective effect on sperm production and contributes to the maturation of spermatocytes. The experimental results of LDH were consistent with the histopathological changes observed in the ST of the testes, further supporting the previously reported association between LDH and the maintenance of testicular tissue stability [53]. These results suggestively describe variations in sperm quality and highlight the importance of LDH in sperm metabolism. Additionally, previous studies described the significance of LDH in facilitating the glycolysis and glucose metabolism of spermatogonia cells [54,55]. In this present study, CSE supplementation successfully restored the LDH activity.

In the presence of stress, specific bacteria or bacterial molecules, activation of the inflammasome complex leads to the generation of active proinflammatory cytokines, particularly IL-1β, exerting effects on diverse neurological, intestinal homeostatic, and inflammatory conditions [56]. Moreover, oxidative stress-induced disruptions in the intestinal barrier allow for the transit of cytokines into the bloodstream, activating TLR4 and the production of inflammatory cytokines. TLRs play a role in bacterial communication and initiate cytokine responses in neurons [57]. Simultaneously, activation of vagal afferent neurons augments IL-1β transcript levels in the hippocampus and elicits IL-1β synthesis in the brain, exerting a direct influence on the central nervous system (CNS) and triggering the hypothalamic–pituitary axis [58]. Additionally, the peripheral release of inflammatory factors enhances blood–brain barrier permeability, enabling their direct impact on the brain [57].

In the present study, substantial impairment of the normal colon structure, widespread ulceration, and inflammation affecting all layers of the gut wall were observed during PS-NPs exposure. A previous study reported that PS-NPs induced intestinal barrier dysfunction through oxidative stress and inflammation [59]. Significant microhemorrhages, edema, and leukocyte infiltration were also demonstrated, resulting in damage to goblet cells and crypt enlargement due to inflammation. As intestinal epithelial cells mutate, deteriorate, or disappear, epithelial permeability increases, allowing bacteria, endotoxins, and macromolecules to enter the systemic circulation [60]. Disruption of the intestinal barrier triggers IL-1β release, activating Toll-like receptor 4 (TLR4) and provoking a cytokine response. Vagus nerve (VN) activation via TLR4 leads to IL-1β synthesis in the brain, ultimately affecting the HPG axis and causing reproductive damage [57,58]. Exposure to PS-NPs induces intestinal damage and triggers the release of pro-inflammatory cytokines. The neutrophilic inflammatory infiltrate is moderately reduced in CSE-treated colonic specimens, which show a remarkable restorative effect. There is scant evidence of dilated crypts and goblet cell depletion in most of these samples; however, mucin replenishment is observed, characterized by the restoration of the epithelial layer and a decrease in ulceration. Intestinal epithelial improvement and enhanced intimate connection of the intestinal mucosal epithelium may be related to intestinal flora that maintains the intestinal barrier functioning [61]. These findings demonstrate that the antioxidant properties of CSE effectively protect the intestinal barrier, likely due to the presence of bioactive compounds derived from EE. A previous study reported that bioactive compounds, including phenolic acids, reduce intestinal damage and improve intestinal barrier integrity [62].

An association between increased *Bacteroides* and decreased *Lactobacillus* and *Firmicutes* has also been observed with gut inflammation in this study. Inflammatory signals originating from the gut, specifically IL-1β and IL-6, activate vagal afferent neurons, and initiating the transmission of inflammatory messages to the brain and engaging the HPG axis, which plays a role in neuroendocrine regulation [63]. Importantly, IL-1β and IL-6 have been associated with hypothalamic modulation, leading to the disruption of normal testicular function. Moreover, administration of CSE after PS-NPs exposure exhibits a significant mitigating effect on the inflammatory response by effectively reducing the levels of inflammatory cytokines, including IL-1β, IL-6, and TNF-α. These findings highlight the potent anti-inflammatory properties of CSE, which may contribute to its ability to alleviate the detrimental effects caused by PS-NPs exposure.

In the gastrointestinal tract, specific cells, including enteroendocrine cells (EECs) and enteric neurons, synthesize and release 5-HT [64]. 5-HT molecules traverse the intestinal wall and nerve fibers, entering the bloodstream to impact neuronal activity and neurotransmission in the brain [65]. Various 5-HT receptors, such as the 5-HT1A receptor, regulate 5-HT activity in the brain [66]. Thus, gut-produced 5-HT can influence brain function. This communication along the gut–brain axis is an important interaction mechanism between the gut and the brain. In this study, male rats subjected to CSE treatment exhibited a significant increase in both colon and brain 5-HT levels compared to the PS-NPs group, along with alterations in the expression of 5-HT1A receptor in the brain. These findings reveal the regulatory effects of CSE on 5-HT levels in the gut and brain, as well as its impact on 5-HT1A receptors. The results may explain the influence of CSE on the gut–brain axis and its potential benefits in mitigating the adverse effects caused by PS-NPs exposure. We hypothesized that CSE also upregulates tight junction proteins such as Zonula occludens-1 (ZO-1) and Occludin, resulting in increased stability of the mechanical barrier [67]. A previous study has reported that *Echinacea purpurea* upregulates the expression of tight junction proteins, including ZO-1, Claudin-1, and Occludin [68]. Previous studies have also reported that key tight junction proteins, including ZO-1, Occludin, and Claudin-1, contribute to forming a selective permeability barrier that facilitates nutrient absorption [69,70].

Overall, this study indicates that CSE supplementation regulates the gut–brain–reproduction axis, which represents a complex and bidirectional communication network between the gastrointestinal, central nervous, and reproductive systems. Previous studies have reported that the gut microbiota plays a critical role in mediating physiological processes that affect brain function and reproductive health, largely through mechanisms involving immune modulation, metabolic signaling, and neurotransmitter regulation [71,72]. Additionally, an imbalance in gut microbial composition has been closely linked to intestinal inflammation and systemic immune activation. Studies have demonstrated that an overgrowth of pro-inflammatory bacteria or a reduction in anti-inflammatory species can trigger an inflammatory response in the gut [73,74]. Mucosal biopsies have also revealed a significant reduction in the Firmicutes/Bacteroidetes (F/B) ratio in IBD samples, indicating that such an alteration in the F/B ratio is associated with changes in energy metabolism [75]. A previous study also reported that the gut microbiota plays an essential role in reproductive health by influencing hormonal balance, immune responses, and inflammation. Dysbiosis has been implicated in a range of reproductive disorders, including endometriosis and infertility [74].

## 4. Materials and Methods

### 4.1. Materials

Polystyrene Nanoplastics (PS-NPs) were purchased from Yaohong Biotechnology Co., Ltd., (New Taipei City, Taiwan; Cat. No. DNM-P004). The sodium silicate was purchased from Cheng Yi Chemical Co., Ltd. (Taipei, Taiwan). The chitosan powder was purchased from Lytone Enterprise, Inc. (Taipei, Taiwan; degree of deacetylation = 81%, molecular weight = 200 kDa). Heparin, trichloroacetic acid, and thiobarbituric acid were purchased from Sigma-Aldrich (St. Louis, MO, USA). Alanine aminotransferase (ALT), aspartate aminotransferase (AST), and glutathione peroxidase (GPx) assay kits were purchased from Randox Laboratories (Crumlin, County Antrim, UK). ELISA kits for tumor necrosis factor-α (TNF- α, Cat. No. ab100785), interleukin-1β (IL-1β, Cat. No. ab100772), and IL-6 (Cat. No. ab100768) were purchased from Abcam Ltd. (Cambridge, MA, USA). Kiss1 receptor (Cat. No. MBS MBS2021161), gonadotropin-releasing hormone (GnRH, Cat. No. MBS762089), testosterone (Cat. No. MBS9424769), and luteinizing hormone (LH, Cat. No. MBS764675), 5-Hydroxytryptamine (5-HT, Cat. No. MBS70267), and 5-hydroxytryptamine receptor 1A (5-HT1A, Cat. No. MBS1604572) were purchased from MyBioSource (San Diego, CA, USA). The DNeasy^®^ PowerSoil^®^ Pro Kit (Cat. No. 47014) was purchased from QIAGEN (Hilden, Germany).

### 4.2. Preparation of Polystyrene Nanoplastics (PS-NPs) Suspension

Polystyrene Nanoplastics (PS-NPs) was purchased from Yaohong Biotechnology Co., Ltd. [size = 0.4–0.6 μm, concentration = 5% *w*/*v*, the number of particles = 1.14 × 10^12^ particles/mL]. The particles were dispersed in deionized water (PS-NPs 5 mg/kg solids suspension) and then ultrasonically treated for 30 min to evenly disperse the plastic particles. The suspensions were freshly prepared every day and were vortexed before each use to prevent aggregation [76].

### 4.3. Preparation of Chitosan-Silica Nanoparticle (CS)

Chitosan-silica nanoparticles (CSs) were prepared by mixing silicate solution and chitosan solution according to the previous report [25]. The silicate solution (0.7% *w*/*w*, pH = 5.6) was prepared by dissolving sodium silicate in 0.05 M sodium acetate solution. The chitosan solution (0.7% *w*/*w*, pH = 5.6) was obtained from dispersing chitosan in 0.5 M acetic acid solution. Chitosan and silica were mixed at a ratio of 10:1. The mixture was stirred homogeneously at room temperature for 1 h and left for 4 h. Next, the mixture was centrifuged at 9600 rpm at 15 °C for 20 min and the precipitates were freeze-dried. The acquired products were chitosan-silica nanoparticles.

### 4.4. Preparation of Echinacea Purpurea Extract Loaded Chitosan-Silica Nanoparticle (CSE)

*Echinacea purpurea* was purchased from Taiwan Direct Biotechnology Corp., Taoyuan, Taiwan, and a voucher specimen was deposited. *Echinacea purpurea* was dried and grounded. Then, 70% ethanol was added and stirred at 40 °C for 24 h to obtain the crude extract. The solution was filtered and freeze-dried (Freeze drying system FD 4.5 12XL, Kingmech Co., Ltd., Taipei, Taiwan). The samples were kept overnight at a −80 °C refrigerator before lyophilization. The acquired extraction powder yields approximately 10% of *Echinacea purpurea* (flower: stalk and leaf: root = 2:7:1).

The encapsulation was performed according to a previously reported method [25] with some modifications. The silicate solution (0.7% *w*/*w*, pH = 5.6) was prepared by dissolving sodium silicate in 0.05 M sodium acetate solution. Chitosan solution (0.7% *w*/*w*, pH = 5.6) was obtained from dispersing chitosan in 0.5 M acetic acid solution. Silicate solution, chitosan solution, and *Echinacea purpurea* ethanol extract (EE) were mixed with a ratio of 10: 1:1. The mixture was stirred homogeneously at room temperature for 1 h and left for 4 h. Next, the mixture was centrifuged at 9600 rpm at 15 °C for 20 min. The precipitates were chitosan-silica-*Echinacea Purpurea* extracts composite nanoparticles (CSE). The obtained material was collected in screw-capped tubes and freezed immediately in liquid nitrogen or stored at −80 °C.

### 4.5. Fourier-Transform Infrared (FTIR) Spectroscopy

The FTIR experiments were conducted using a Bruker TENSOR II FTIR spectrometer (Billerica, MA, USA). To prepare the samples for analysis, CS, CSE, and EE were freeze-dried and ground into a powder before being introduced to the spectrometer. A total of 64 scans were performed for each spectrum in the wavenumber range of 650 to 4000 cm^−1^ with a resolution of 4 cm^−1^.

### 4.6. Dynamic Light Scattering (DLS) Analysis

To prepare the aqueous suspensions at 0.2% (*w*/*w*), the CS and CSE samples were dispersed in deionized water and sonicated for 10 min to prevent nanoparticle aggregation. Then, 700 μL of the suspension was transferred to a cuvette and the average particle size and zeta potential of the nanoparticles were analyzed using a DLS instrument at 25 °C.

### 4.7. Animal Study

Forty-two healthy 7-week-old male Sprague-Dawley (SD) rats (BioLASCO Taiwan Co., Ltd., Yilan, Taiwan) were kept under standard laboratory conditions (light/dark cycles of 12 h/12 h, the humidity of 40–60% and constant temperature of 20 ± 2 °C) in polypropylene cages and fed standard chow diets (PMI Nutrition International, Inc., USA). Food and water were provided ad libitum. The Institutional Animal Care and Use Committee, College of Life Science, National Taiwan Ocean University, evaluated and approved (IACUC Approved Number 111022) the study protocols to use laboratory animals for this study, following Directive 2010/63/EU guidelines. Briefly, rats were acclimatized for a week and then randomly divided into six groups (n = 7): Control group (without any treatment), PS-NPs group (5 mg/kg PS-NPs), CS group (5 mg/kg PS-NPs + 372 mg/kg CS), CSE1X group (5 mg/kg PS-NPs + 93 mg/kg CSE), CSE2X group (5 mg/kg PS-NPs + 186 mg/kg CSE), and CSE4X group (5 mg/kg PS-NPs + 372 mg/kg CSE). Animal body weights were measured once a week during the whole experiment. All groups except the Control group were orally gavaged with 5 mg/kg PS-NPs per day for 8 weeks to induce the reproductive injury. The CSE doses were chosen according to the previous study [52]. Furthermore, the Control and PS-NPs groups were orally gavaged and administered by saline while other groups received samples orally for 8 weeks. The CS and CSE were dissolved in distilled water (dH_2_O) to make the concentration. All animals were sacrificed after 8 weeks of treatments.

#### 4.7.1. Blood Sample Collection

Blood samples were collected and centrifuged at 3000× *g* at 4 °C for 15 min. The anticoagulant used in this study was heparin. The testis, hypothalamus, brain, and gut were also taken for histological evaluation. All the samples were immersed in 10% formaldehyde or stored at −80 °C for further analysis.

#### 4.7.2. 16S rRNA Gene Sequencing Analysis

At least two fecal pellets were collected from each rat a day before sacrifice and subsequently stored at −80 °C. Metagenomic analysis was performed on four biological samples randomly selected from each group. Microbial genomic DNA was extracted from the fecal pellets using the DNeasy^®^ PowerSoil^®^ Pro Kit (Qiagen), following the manufacturer’s protocols, and stored at −80 °C for further experimentation. The quality and quantity of the extracted DNA were assessed by determining the ratios of absorbance at 260 nm/280 nm and 260 nm/230 nm, respectively, according to a previous method [77]. The samples were then submitted to the Marine Center of National Taiwan Ocean University (Keelung City, Taiwan), where PCR amplification of the 16S rRNA gene was performed, followed by analysis of the microbial communities within each respective group [78].

#### 4.7.3. Histopathological Analysis

Histopathological analysis was conducted on the testicular and colon to provide a standardized and quantitative assessment of the severity of pathological changes in these tissues. The testis and distal colon were immersed in formalin and sent to Rapid Science Co., Ltd. (Brooklyn, NY, USA) for paraffin-embedding and hematoxylin and eosin (H&E) staining. For the testicular tissue sections, images were analyzed using ImageJ software (ver. 1.54c) to evaluate pathological changes in the seminiferous tubules (ST), including measurements of ST diameter, epithelial thickness, area of seminiferous tubules, and area of seminiferous tubule lumen as a percentage of the ST. In the case of colonic tissue, histology was scored using a previously described scoring system that assessed the extent of damage to the colonic mucosa. Scores ranged from normal mucosa (Score 0) to severe erosions and marked inflammatory cell infiltration (Score 4), with intermediate scores indicating partial damage to the colonic crypts: loss of one-third of the crypts (Score 1) and loss of two-thirds of the crypts (Score 2); as well as mild inflammatory cell infiltration and epithelial changes (Score 3) [79]. These histopathological analyses offer an objective and accurate approach to evaluating pathological changes in the testicular and colonic tissues.

#### 4.7.4. Determination of Malondialdehyde (MDA) Level

An aliquot of 100 μL (plasma and tissue homogenates) was mixed with 200 μL of malondialdehyde (MDA) reagent. MDA reagent was a mixture of 15% (*w*/*v*) trichloroacetic acid in 0.25 N HCl and 0.375% (*w*/*v*) thiobarbituric acid in 0.25 N HCl. The above-said solution was kept in a dry bath at 100 °C for 15 min. Later, 300 μL n-butanol was added to the solution, mixed well, and centrifuged at 1500× *g* for 10 min. Then, 100 μL supernatant was taken, and the absorbance was measured at 532 nm. In total, 5 nmol of 1, 1, 3, 3-tetramethoxypropane was used as the standard. The MDA level was determined according to the equation [80]:

MDA (nmol/mL) = [(A _sample_ − A _blank_)/(A _standard_ − A _blank_)] × 5 nmol

#### 4.7.5. Determination of Nitric Oxide (NO) Level

The nitric oxide (NO) level was evaluated by the Griess reagent method. The Griess reagent was a mixture of 50 μL sulfanilamide (SUL) solution (0.1 g SUL in 10 mL 5% phosphoric acid) and 50 μL N-(1-naphthyl) ethylenediamine dihydrochloride (NED) solution (0.01 g NED in 10 mL deionized water). The cell concentration was first adjusted to 1 × 10^6^ cells/well. A 100 μL sample (plasma, tissue homogenates, and sperm cell) was mixed with an equal amount of Griess reagent in a 96-well plate and incubated for 10 min at 37 °C in the dark. Later, the absorbance was measured at 540 nm. Sodium nitrate was taken as the standard. The NO level was quantified by plotting the standard curve [81].

#### 4.7.6. Determination of Biomarkers Related to Liver Damage, Pro-Inflammatory Cytokines, Enzymatic Antioxidant, and Reproductive Function

The levels of biomarkers related to liver damage (ALT and AST), pro-inflammatory cytokines (TNF-α, IL-1β, and IL-6), antioxidant enzyme (GPx), sperm metabolism-related enzyme (LDH), reproductive endocrine (Kiss1R, GnRH, LH, and testosterone), and neurotransmitter and its receptor (5-HT and 5-HT1A) in male rats were measured using appropriate assay kits. All procedures were carried out following the manufacturer’s instructions.

### 4.8. Statistical Analysis

The data are presented as mean ± standard deviation (SD). Statistical analysis was performed using GraphPad Prism 9 and SPSS 22.0 (Statistical Product and Service Solutions). One-way analysis of variance (ANOVA) followed by Tukey’s multiple comparison test was used to evaluate differences, with *p* < 0.05 considered statistically significant.

## 5. Conclusions

Our study focused on improving the gut microbial composition and regulating the inflammation, along with the protection of gut–brain axis. Consumption of polystyrene microplastics significantly impacts reproductive damage by affecting the hypothalamic–pituitary–testicular axis and causing subsequent damage to spermatozoa and the testis. In this study, we investigated the effect of EE in ameliorating this reproductive damage. The presence of phytochemicals within the EE, such as phenolic acid, cichoric acid, and isobutylamides, contributes anti-inflammatory activity, antimicrobial and antioxidant properties. The addition of EE into chitosan-silica nanoparticles addresses the limitations of EE, including bitter taste, low aqueous solubility, and poor oral bioavailability. Our findings open a new pathway to recover reproductive health by improving gut microbiota dysbiosis, reducing inflammation, and modulating the HPG axis. An increase in ALT and AST levels is associated with liver dysfunction, and our findings demonstrated reduced levels of ALT and AST after CSE treatment. Additionally, we observed a decrease in oxidative damage through reducing cytokine levels and regulating neurotransmitter levels.

## Data Availability

The data that support the plots within this paper and other findings of this study are available from the corresponding authors upon reasonable request.
